# Past oral contraceptive use and self-reported high blood pressure in postmenopausal women

**DOI:** 10.1186/s12889-015-1392-3

**Published:** 2015-01-31

**Authors:** Christine L Chiu, Joanne M Lind

**Affiliations:** School of Medicine, University of Western Sydney, Locked Bag 1797, Penrith, NSW 2751 Australia

**Keywords:** Hormonal contraception, High blood pressure, Postmenopausal women

## Abstract

**Background:**

Studies have reported current hormonal contraceptive use is associated with adverse cardiovascular outcomes, including high blood pressure. The aim of this study was to determine the association between past hormonal contraception use and high blood pressure in Australian postmenopausal women.

**Methods:**

Women were recruited from the *45 and Up Study*, an observational cross-sectional study, conducted from February 2006 to December 2009, NSW Australia. All of the variables used in this study were derived from self-reported data. These women reported being postmenopausal, having an intact uterus, and had given birth to one or more children. Odds ratios and 99% confidence intervals for the association between past hormonal contraceptive use and current treatment for high blood pressure, stratified by current age (<58 yrs, 58–66 yrs, and ≥67 yrs) were estimated using logistic regression, adjusted for income, country of origin, BMI, smoking, alcohol, exercise, family history of high blood pressure, menopausal hormone therapy use, number of children, whether they breastfed, and age of menopause.

**Results:**

A total of 34,289 women were included in the study. No association between past hormonal contraception use and odds of having high blood pressure were seen in any of the age groups (<58 yrs: odds ratio (OR) 1.1, 99% confidence interval (CI) 0.8 to 1.5, p = 0.36; 58–66 yrs: OR 0.9, 99% CI 0.7 to 1.1, p = 0.11; and ≥67 yrs: OR 0.9, 99% CI 0.8 to 1.0. p = 0.06). In women with a history of hormonal contraception use, no association between duration of hormonal contraception use and high blood pressure was observed.

**Conclusions:**

Past hormonal contraception use and duration of use is not associated with high blood pressure in postmenopausal women.

## Background

Hormonal contraceptives are the most commonly used method of birth control worldwide. Since their introduction in the 1960s, adverse cardiovascular outcomes, including increased risk of myocardial infarction [1], stroke [1,2], venous thrombosis [3] and high blood pressure [4-6] have been reported. These studies have predominantly looked at current hormonal contraception use in younger women.

Hypertension (high blood pressure) is a leading cause of morbidity and mortality in postmenopausal women. Endogenous estrogens are thought to protect women against vascular disease and atherosclerosis [7], while exogenous estrogens have been associated with increased risk of stroke and high blood pressure [8,9]. The Nurse’s Health Study investigated the effect of long term hormonal contraception use on cardiovascular disease mortality, and found no association between increasing duration of past hormonal contraceptive use and total mortality related to cardiovascular disease [10].

The present study investigated the relationship between past hormonal contraception use and likelihood of high blood pressure in Australian postmenopausal women. The aim of this study was to determine the association between past hormonal contraception use and the likelihood of having high blood pressure.

## Methods

Data was obtained from women who were recruited from the *45 and Up Study*. The methods for the *45 and Up Study* have been described elsewhere [11]. Briefly, the *45 and Up Study* is a large scale cohort study of healthy ageing that involves men and women aged 45 years and over from the general population of New South Wales, Australia. Individuals were sampled from the Medicare Australia database. Study recruitment started in January 2006 and was completed in April 2009. The *45 and Up Study* received ethics approval from the University of NSW Human Ethics Committee (HREC 10186) and the current study was approved by the University of Western Sydney Human Research Ethics Committee (H8561).

**Figure 1:**
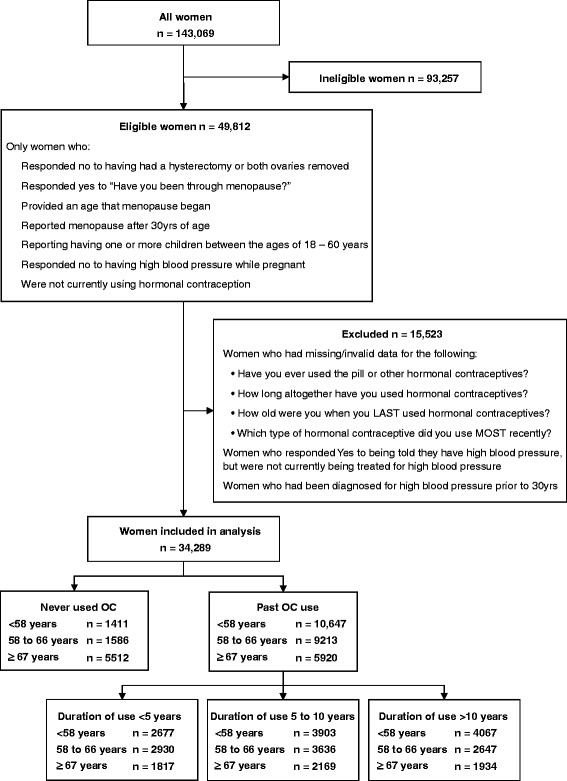
**Participants included in the study.**

All of the variables used in this study were derived from self-reported data obtained from the *45 and Up Study* baseline questionnaire (available at www.45andUp.org.au). Women were included in this study if: they fulfilled the selection criteria outlined in Figure 1. Regarding hormonal contraception use, women were asked “Have you ever used the pill or other hormonal contraception?” and “If Yes, for how long altogether have you used hormonal contraception? (in years)” and “If Yes, how old were you when you LAST used hormonal contraceptives?”. Women were also asked “Which type of pill or other hormonal contraceptive did you use MOST RECENTLY; combined pill; progesterone only; Depo Provera; contraceptive implant; or do not know?”. Examples of common brands were given with each type. Progesterone only, Depo Provera and contraceptive implant were combined as the progestin group. Women whose current age was greater than the age they reported last using hormonal contraception were classified as past users. Hormonal contraception use was analysed as a dichotomous variable (past use/never). Duration of use in years was analysed as a categorical variable (never, <5 years, 5 to 10 years, and >10 years). Women with missing or invalid data for these variables were excluded. Women were identified as having high blood pressure if they answered “Yes” to the question “In the last month have you been treated for: high blood pressure”.

Odds ratios and 99% confidence intervals were estimated using logistic regression. Both crude and adjusted odds ratios were calculated and descriptions refer to adjusted odds ratios unless otherwise specified. Odds ratios were adjusted for demographic and lifestyle factors that were significantly associated with treatment for high blood pressure in univariate analysis. Namely, income, country of origin, BMI, smoking, alcohol, exercise, family history of high blood pressure, menopausal hormone therapy use, number of children, whether they breastfed, and age of menopause, with additional categories for missing values (Table 1). For ‘Country of birth’, participants were classified according to whether they had been born in Australia or born in a country other than Australia. Physical activity levels were assessed using questions from the Active Australia Survey Vigorous activity as previously described [8]. Smoking was classified as current, past or never, according to the questions “Have you ever been a regular smoker?” and if yes, “Are you a regular smoker now?”. Consumption of alcohol was classified into the groups shown in Table 1 based on the question “About how many alcoholic drinks do you have each week?”.

**Table 1:** **Socio-demographic factors and health risk factors associated with hormonal contraception use**

**Characteristics**		**n (% column)**	**% HC**	**Odds ratio** ^**†**^ **(99% CI)**
**Country of origin**	Australia	25,664 (75)	79	1.0
	Other	8,348 (24)	64	0.5 (0.4 to 0.5)
**Income**	<$30 K	10,294 (30)	64	1.0
	$30 K – $70 K	8687 (25)	83	2.2 (2.1 to 2.4)
	$70 K+	7,037 (21)	89	3.7 (3.4 to 4.0)
	Did not disclose	8,271 (24)	69	1.2 (1.1 to 1.3)
**Body Mass Index (BMI)**	<25	15,561 (45)	75	1.0
	25 to 29	10,410 (30)	76	1.1 (1.1 to 1.2)
	+30	5,731 (17)	77	1.4 (1.3 to 1.5)
**Family History HBP**	No	16,994 (50)	71	1.0
	Yes	17,295 (50)	79	1.3 (1.3 to 1.4)
**Smoking status**	Never	22,626 (66)	72	1.0
	Past	9,483 (28)	82	1.5 (1.4 to 1.6)
	Current	2,040 (6)	81	1.9 (1.7 to 2.2)
**Alcohol (drinks/week)**	0	13,137 (38)	65	1.0
	1-5	9,276 (27)	81	1.7 (1.6 to 1.8)
	6-10	6,889 (20)	82	1.7 (1.6 to 1.9)
	11+	4,335 (13)	86	1.9 (1.7 to 2.1)
**Physical activity**	Insufficient	10,071 (29)	66	1.0
	Sufficient	24,218 (71)	79	1.6 (1.5 to 1.7)
**MHT use**	No	21,531 (63)	42	1.0
	Yes	12,422 (36)	85	2.3 (2.2 to 2.4)
**Number of children**	1	3,580 (10)	71	1.0
	2	13,772 (40)	82	1.5 (1.4 to 1.7)
	3	10,184 (30)	78	1.3 (1.1 to 1.4)
	4+	6,753 (20)	63	0.7 (0.6 to 0.8)
**Breastfed**	No	3,986 (12)	72	1.0
	Yes	29,889 (87)	76	1.1 (1.0 to 1.1)
**Age at menopause (years)**	mean ± SD	49.9 ± 4.5		−0.01(−0.01 to −0.001)*

HC = Hormonal Contraceptive; OR = Odds Ratio; CI = Confidence Interval. % HC is the percentage of women who responded yes to having ever used hormonal contraception. Percentages do not consistently total to 100% due to missing values. ^†^Adjusted for age, country of origin, income level, body mass index, family history of high blood pressure, smoking status, alcohol consumption, physical activity, menopausal hormone therapy (MHT) use, number of children, whether they breastfed, age at menopause. *Linear variable, therefore beta value and 99% CI reported.

Tests for interactions between covariates and past hormonal contraception use, with odds for high blood pressure were performed. A significant interaction between age and past hormonal contraception use with having high blood pressure was observed (p < 0.001). As a result, women were stratified according to age and divided into tertiles (<58 yrs, 58–66 yrs, and ≥67 yrs). Odds ratios were calculated for each age group. All statistical tests were two-sided, using a significance level of p < 0.01 to partially account for multiple testing issues [12]. Conclusions were drawn based on both significance and the effect size.

## Results

A total of 34,289 women were included in the study (Figure 1) of which 75% reported past hormonal contraceptive use and 21% reported current treatment for high blood pressure. These women were postmenopausal, reported having an intact uterus, had given birth, and had not had high blood pressure while pregnant. Past hormonal contraception use was lowest in women who were born prior to 1930 and highest in women who were born during the 1950s, with a large percentage (85 to 88%) of women that were born between 1940 and 1960 reporting previous hormonal contraception use. Women who had previously used hormonal contraceptives were significantly younger than women who had never used hormonal contraceptives, 61.0 years compared to 71.4 years, respectively (β -10.4; CI 99% -10.7 to −10.1; p < 0.001).

Demographic and lifestyle characteristics of past users compared to women who have never used hormonal contraceptives are outlined in Table 1. Women who reported being born in Australia, had a higher income, were physically active, were current smokers, reported drinking alcohol, had 2 or 3 children, and had a history of menopausal hormone therapy use were more likely to have used hormonal contraceptives, compared to women who were not born in Australia, had a lower incomes, were non-smokers and non-drinkers, had not used menopausal hormone therapy, and who had only one child, respectively.

In all age groups no association between previous hormonal contraceptive use and high blood pressure was observed (Table 2). Similarly, duration of hormonal contraception use was not associated with odds for high blood pressure in any age group, when compared to never users. Within women who reported hormonal contraception use, there was no difference in odds for high blood pressure between those that had used hormonal contraception for less than 5 years and those that had used it for 5 to 10 years, or greater than 10 years (Table 3).

**Table 2:** **The odds for having high blood pressure in postmenopausal women who had previously used hormonal contraceptives compared with women who have never used hormonal contraceptives, stratified by current age**

**Current age**	**HC use**	**n**	**% HBP**	**Unadjusted OR (99% CI)**	**Unadjusted P value**	**Adjusted** ^**†**^ **OR (99% CI)**	**Adjusted P value**
<58 yrs	Never	1411	8.9	1.0		1.0	
	Past use	10647	10.6	1.2 (0.9 to 1.6)	0.05	1.1 (0.8 to 1.5)	0.36
58 to 66 yrs	Never	1586	20.7	1.0		1.0	
	Past use	9213	19.6	0.9 (0.8 to 1.1)	0.29	0.9 (0.7 to 1.1)	0.11
≥67 yrs	Never	5512	33.2	1.0		1.0	
	Past use	5920	33.4	1.0 (0.9 to 1.1)	0.86	0.9 (0.8 to 1.0)	0.06

HC = Hormonal Contraceptive; HBP = High Blood Pressure; OR = Odds Ratio; CI = Confidence Interval. ^†^Adjusted for income, country of origin, BMI, smoking, alcohol, exercise, family history of high blood pressure, menopausal hormone therapy use, number of children, whether they breastfed, and age of menopause.

**Table 3:** **The odds of having high blood pressure depending on duration of hormonal contraception use compared to never use, stratified by current age**

**Current age**	**HC use**	**n**	**% HBP**	**OR (99% CI)** ^**†**^	**P value**
<58 years	Never	1411	8.9	1.0	
	<5 years	2677	9.7	1.0 (0.7 to 1.4)	0.99
	5 to 10 years	3903	10.8	1.2 (0.9 to 1.6)	0.24
	>10 years	4067	11.0	1.2 (0.9 to 1.6)	0.22
58 to 66 years	Never	1586	20.7	1.0	
	<5 years	2930	18.7	0.9 (0.7 to 1.1)	0.05
	5 to 10 years	3636	19.7	0.9 (0.7 to 1.1)	0.17
	>10 years	2647	20.4	0.9 (0.8 to 1.2)	0.42
≥67 years	Never	5512	33.2	1.0	
	<5 years	1817	33.9	1.0 (0.8 to 1.1)	0.38
	5 to 10 years	2169	33.7	0.9 (0.8 to 1.1)	0.23
	>10 years	1934	32.5	0.9 (0.8 to 1.0)	0.04

HBP = High Blood Pressure; OR = Odds Ratio; CI = Confidence Interval. ^†^Adjusted for income, country of origin, BMI, smoking, alcohol, exercise, family history of high blood pressure, menopausal hormone therapy use, number of children, whether they breastfed, and age of menopause.

Of the women who had used hormonal contraception, the combined pill was the most common type of hormonal contraception used by women in all age groups, with over half of women reported having last used the combined pill compared to the less than 15% of women who reported last using a progestin based formulation.

## Discussion

This study found past hormonal contraception use was not associated with subsequent high blood pressure in a cohort of postmenopausal Australian women. Additionally, duration of hormonal contraception used was not associated with odds of having high blood pressure. Women who were born prior to 1930 were significantly less likely to have used hormonal contraception. Oral hormonal contraception was not available in Australia until 1961 and was not widely accessible until the early 1970s. By this time, women born prior to 1930 most likely did not require contraception.

Studies have repeatedly reported increases in blood pressure with current hormonal contraception use [4,6,13-15]. These studies also demonstrated that this effect quickly diminishes once hormonal contraception is stopped [6,13,15,16]. Very few studies have focused on past hormonal contraception use and risk of subsequent high blood pressure. The Nurse’s Health Study (NHS) reported past hormonal contraception use, and duration of use, did not increase a woman’s risk of cardiovascular disease, stroke, or coronary artery disease [10,17,18]. A secondary study (NHS II) study found past users had a slight but not significant increase in risk for hypertension [19].

The women included in this study represent a cohort of women who were exposed to early generations of hormonal contraception, and the results of this study likely reflect associations with higher dose formulations. The later generations of hormonal contraceptives contain lower doses of estrogen and progestin, as well as newer combinations of the hormones [20]. Recent studies using lower dosage contraceptives have shown a reduction in the size of the effect on risk of cardiovascular disease [21]. As formulations and delivery methods change, the effect of long term hormonal contraception use on risk of subsequent high blood pressure may change and would be of interest to both the women receiving treatment and their prescribing clinician.

A limitation of the study was that it analysed cross-sectional observational data, and as a result, the cause could not be established. The study also relied on self-reported data which is prone to potential recall bias, and may lead to under-reporting or over-reporting of diagnosis of high blood pressure, menopause status, and duration of hormonal contraception use. Furthermore factors that are known to influence the incidence of high blood pressure, including type and dosage of hormonal contraception could not be determined. The hormone type that was most recently used was reported, however it is probable that in the time preceding data collection, women may have used different hormonal contraceptives with varying combinations of estrogen and progestin dosage. Additionally, women who have high blood pressure at the time of requiring contraception are often advised against hormonal contraception use, as high blood pressure is a contraindication for hormonal contraceptive use. In an attempt to overcome this, we excluded women who had been diagnosed with high blood pressure prior to 30 years of age as it is unlikely the cause of high blood pressure in these women is a result of exposure to hormonal contraception.

## Conclusions

This study found past hormonal contraceptive use, and duration of use, was not associated with high blood pressure in Australian postmenopausal women.

### Ethics approval

The *45 and Up Study* received ethics approval from the University of NSW Human Ethics Committee (HREC 10186) and the current study was approved by the University of Western Sydney Human Research Ethics Committee (H8561).
